# The risk factors of procedure-related complications after amniocentesis in twin pregnancies: a retrospective analysis

**DOI:** 10.1186/s12884-023-05884-z

**Published:** 2023-08-15

**Authors:** Xijing Liu, Jiamin Wang, Wanying Luo, Qiyi Wang, Zhushu Liu, He Wang, Shanling Liu, Ting Hu

**Affiliations:** 1 Department of Medical Genetics, West China Second University Hospital, Sichuan University, No. 20, Section 3, Renminnan Road, Chengdu, 610041 Sichuan China; 2 Department of Obstetrics and Gynecology, West China Second University Hospital, Sichuan University, Chengdu, 610041 Sichuan China; 3 Key Laboratory of Birth Defects and Related Diseases of Women and Children (Sichuan University), Ministry of Education, Chengdu, 610041 China

**Keywords:** Amniocentesis, Chorionicity, Procedure-related complications, Twin pregnancies

## Abstract

**Background:**

There is an increasing demand for prenatal diagnostic testing in twin pregnancies, however, anecdotally there is a higher incidence of procedure-related complications after amniocentesis than that in singleton pregnancies. There is a paucity of data regarding risk factors of amniocentesis in twin pregnancies.

**Methods:**

Women with twin pregnancies who underwent amniocentesis between January 2016 and December 2020 were enrolled in this retrospective study. Procedure-related complications including spontaneous miscarriage, intrauterine fetal death, spontaneous preterm delivery, preterm premature rupture of membranes, and placental abruption in one or both fetuses after amniocentesis were assessed. Meanwhile, potential risk factors related to amniocentesis including chorionicity, gestational age, conception, number of needle insertions, parity, history of miscarriage, indications, and pregnancy-related complications (pregnancy-induced hypertension and gestational diabetes) were also recorded.

**Results:**

A total of 811 women with twin pregnancies underwent amniocentesis were included, with a procedure-related complications rate of 3.83%. Risk factors associated with increased risk of procedure-related complications after amniocentesis in twin pregnancies were chorionicity (adjusted odds ratio [aOR]: 4.06), gestational age at the procedure (aOR: 2.76), and numbers of needle insertions (aOR: 3.26). In the monochorionic twin pregnancy, hemorrhage during this pregnancy (aOR: 12.01), polyhydramnios (aOR: 5.03), and numbers of needle insertions (aOR: 3.15) were risk factors after amniocentesis. In the dichorionic twin pregnancy, gestational age at the procedure (OR:4.47) affected the risk of procedure-related complications after amniocentesis. In the subgroup of gestational age at the procedure ≤ 24^+ 0^ weeks, risk factors associated with increased risk of procedure-related complications after amniocentesis in twin pregnancies were chorionicity (aOR: 5.14), and numbers of needle insertions (aOR: 3.76).

**Conclusion:**

The procedure-related complications rate is 3.83% in our institution during the study period. The present study has emphasized the significance of certain risk factors for adverse outcome and will be useful in counseling patients with twin pregnancies.

**Supplementary Information:**

The online version contains supplementary material available at 10.1186/s12884-023-05884-z.

## Introduction

The number of twin pregnancies has increased substantially over the last few decades, mainly because of the expanded use of assisted reproductive techniques (ART) and ovulation induction, both of which are also associated with delayed childbearing [1]. Twin pregnancies, especially dizygotic twins, have a higher risk of aneuploidy than singleton pregnancies, in addition to the risk of chromosomal abnormalities due to advanced maternal age (AMA) [1]. In addition, it has been reported that there is an increased rate of structural abnormalities in twins compared with singleton pregnancies and a further increase in the risk of congenital anomalies in monozygotic twins compared with dizygotic twins [2, 3]. Advances in imaging technologies, such as ultrasound and fetal magnetic resonance imaging, also provide more cases in which prenatal diagnosis of fetal genetic disorders is indicated [4].

It has been confirmed that the accuracy of maternal serum screening tests for aneuploidies in twins, using either first- or second-trimester analyte screening, is significantly lower (by at least 15%) than that for singletons [5, 6]. Meanwhile, the sensitivity of non-invasive prenatal testing (NIPT) in twin pregnancies is lower than that in singleton pregnancies, approximately 8.3% for Down syndrome and 20.6% for Edwards syndrome [7]. Thus, there is a need and a demand for prenatal diagnostic testing in twin pregnancies. Amniocentesis is the most frequently used sampling method for prenatal diagnosis and is generally offered after 15-weeks’ gestation [8, 9]. Benefiting from improvements in detection techniques such as molecular genetic testing, amniocentesis can be conducted in advanced gestation and even delayed until the third trimester.

When counseling and consenting a patient for an amniocentesis, one issue that must be mentioned is the risk of “procedure-related complication” referring to spontaneous miscarriage, intrauterine fetal death, spontaneous preterm delivery, preterm premature rupture of membranes, etc. The complication rate from amniocentesis in singleton pregnancies is well established, with the risk of fetal loss ranging from 0.4 to 1.4% [10–13]. A large meta-analysis of 42,716 women who underwent amniocentesis reported that the procedure-related risk of miscarriage was 0.11%, which was much lower than previously published rates [14]. Moreover, an observational study pointed out that the risk factors associated with an increased risk of fetal loss after amniocentesis were maternal age, vaginal spotting and serious bleeding during pregnancy, history of 2nd trimester termination of pregnancy, history of more than three spontaneous or surgical first trimester abortions, and stained amniotic fluid [12].

A higher incidence of procedure-related complications after amniocentesis has been reported in multiple pregnancies. The risks from amniocentesis in twin pregnancies could be higher than that recently published for singleton pregnancies, and more than the empirical risk estimate obtained by simply doubling the loss rate for singleton pregnancies. Two separate meta-analyses identified pregnancy loss rates of 3.07% and 3.5% [15, 16].

However, there is a paucity of data regarding the risk factors for amniocentesis techniques in twin pregnancies, which complicates patient counseling confusing. The present study retrospectively investigated the risk factors for procedure-related complications after amniocentesis in twin pregnancies, with a 4-week follow-up period. Univariate and multivariate analyses were performed to analyze the risk factors contributing to the complication rate, such as chorionic properties, indications, complications, maternal age, and history of miscarriage.

## Methods

### Subjects

This was a single center 5-year retrospective study conducted between January 2016 and December 2020 at West China Second University Hospital, Sichuan University, a tertiary referral center. The inclusion criteria were pregnant women who had two live fetuses and underwent amniocentesis for prenatal diagnosis after 16 + 0 gestational weeks with no history of a vanishing fetus or empty gestational sac in the first trimester. Exclusion criteria were pregnant women who had monoamniotic twin pregnancies, who underwent elective pregnancy termination, who had already undergone chorionic villus sampling, who had amniocentesis performed for assessment of fetal lung maturity, who underwent a selective reduction of triplet pregnancies, or who were diagnosed with twin-twin transfusion syndrome (TTTs), twin anemia polycythemia sequence (TAPS), twin reversed arterial perfusion (TRAP) and selective fetal growth restriction (sFGR).

Detailed information was recorded, including the patient’s demographic data (maternal age, gestational age at amniocentesis, conception methods, indications, medical history, pregnancy complications), details of the procedures (number of needle insertions, color of amniotic fluid, complications during and after the procedures), and chorionicity.

For ART pregnancies, gestational age was calculated from the date of embryo transfer and confirmed by first trimester ultrasound scans. For spontaneously conceived pregnancies, the gestational age was calculated according to an ultrasound examination between 11 + 0 and 13 + 6 weeks of gestation (crown–rump length, 45–84 mm).

Indications for amniocentesis included AMA, abnormal maternal serum screening for aneuploidies, positive NIPT results, abnormal sonographic findings, family or personal history, suspicion of infection, and maternal requests. Pregnancy-related complications include pregnancy-induced hypertension and gestational diabetes. Chorionicity was determined by ultrasonography during the first trimester. Pregnancies were considered monochorionic in the presence of the T-sign and dichorionic in the presence of the λ-sign, or when two separate placental masses were present in the first trimester.

Procedure-related complications that were recorded included spontaneous miscarriage, intrauterine fetal death, spontaneous preterm delivery, preterm premature rupture of membranes, and placental abruption in one or both fetuses after amniocentesis. Pregnancy loss was defined as delivery at less than 24 weeks of gestation. Preterm delivery was defined as delivery at < 37 weeks of gestation. The timeline for procedure-related complications reported in the literature is up to 4 weeks after amniocentesis or 24 − 28 gestation weeks. For our study, the timeline for related complications was defined as four weeks after the procedure.

### Amniocentesis

Amniocentesis was carried out by skilled obstetricians certified by national organization. Additionally, their proficiency is evaluated every two years to ensure consistent high-quality performance. Amniocentesis was performed under continuous ultrasound guidance (Voluson E8; GE Medical Systems, Zipf, Austria) by fetal sonographers who directly assisted with the procedure. Before the procedure, ultrasonography was performed to confirm the number of fetuses, fetal heart rate, fetal position, placental location, and placental umbilical cord insertion site. All procedures were performed as a sterile technique without any local anesthesia, and in a similar manner: a 21-gauge needle (CHI2115; CareFusion, Illinois, USA) was inserted into the amniotic sac of one twin; after discarding the first 1–2 mL fluid to avoid maternal cell contamination, approximately 15 mL of amniotic fluid was aspirated; no dye instillation of 8–10 mL amniotic fluid aspirated from the sac was used to confirm the separation of each sac; once the first needle was withdrawn from the maternal abdomen, a separate needle was inserted into the other sac at a different puncture site to collect amniotic fluid of the second fetus.

The specific procedural variables registered for each patient included the amniotic fluid volume and color, number of needle insertions, and transplacental technique. Women were sent home 30 min after the procedure, and rest for 5–7 days was recommended. All women were recommended amniocentesis using the double-puncture technique for each sac unless they declined, even if a certain diagnosis of monochorionicity was made.

### Statistical analysis

Qualitative variables are presented as absolute and relative frequencies. The normality of quantitative variables was assessed using Q-Q plots and the presentation of these variables includes measures such as mean and standard deviation, or median and interquartile range as appropriate.

Risk factors for procedure-related complications were stratified according to chorionicity, gestational age, conception, number of needle insertions, nulliparity, hemorrhage during this pregnancy, history of miscarriage, amniotic fluid characters, indications, and complications. Univariate logistic regression analysis was used to test the effects of the investigated factors on procedure-related complications. A multivariate logistic regression analysis was conducted with adjustments for potentially confounding effects. Data was modeled using backward stepwise logistic regression analysis with variable removal set at *P* = 0.10 and variable entry set at *P* = 0.05.

Then univariate logistic regression analyses and multivariate logistic regression analyses were used for dichorionic twins and monochorionic twins separately. In addition, a subanalysis according to gestational age at the procedure (≤ 24^+ 0^ vs. > 24^+ 0^ weeks) was also employed consistent above approaches. Odds ratios with 95% confidence intervals (CI) were computed from the results of logistic regression analysis. All *P* values reported are two-tailed. Statistical significance was set at *P* < 0.05, and analyses were conducted using the SPSS statistical software (version 19, SPSS Inc., Chicago, IL, USA).

## Results

Between January 2016 and December 2020, 850 pregnant women with twin pregnancies were underwent amniocentesis in our unit. Thirty-nine cases were excluded according to mentioned criteria, including four cases of monoamniotic twin pregnancies, 14 cases of elective pregnancy termination, 19 cases with a selective reduction of triplet pregnancies, and two cases of TTTs. As a relative short follow-up time, there was no case lost to follow-up. In the study group, the procedures were performed in 592 dichorionic twin pregnancies (73.0%) and 219 monochorionic twin pregnancies (27.0%), with a median gestational age of 21 + 3 weeks, ranging from 16 + 5 to 32 + 5 weeks. Mean maternal age was 31.76 ± 4.76 years, and the portion of AMA (≥ 35) was 68.19% (553/811). Most amniocentesis procedures (79.3%, 634/811) were performed ≤ 24^+ 0^ gestation weeks. Women who conceived through ART comprised 60.91% (494/811). The demographic and clinical data of the study population are presented in Table 1.

**Table 1:** The demographic and clinical data of pregnant women with twin pregnancies underwent amniocentesis

	n (%)
**Chorionicity**	
Dichorionic	592 (73.00)
Monochorionic	219 (27.00)
**Gestational age at the procedure (weeks)**	
≤ 24^+ 0^	643 (79.30)
> 24^+ 0^	168 (20.70)
**Conceived way**	
ART	494 (60.91)
Spontaneous	317 (39.09)
**Numbers of needle insertions**	
2	713 (87.92)
1	98 (12.08)
**Hemorrhage during this pregnancy**	
No	775 (95.56)
Yes	36 (4.44)
**Nulliparity**	
Yes	615 (75.83)
No	196 (24.17)
**Number of miscarriages in the 1st trimester**	
< 2	635 (78.30)
≥ 2	176 (21.70)
**History of miscarriage in the 2nd trimester**	
No	781 (96.30)
Yes	30 (3.70)
**Stained amniotic fluid**	
No	687 (84.71)
Yes	124 (15.29)
**Indication**	
**Age**	
Non-AMA	258 (31.81)
AMA	553 (68.19)
**Structural Abnormality**	
No	562 (69.30)
Yes	249 (30.70)
**Polyhydramnios**	
No	751 (92.60)
Yes	60 (7.40)
**IUGR**	
No	778 (95.93)
Yes	33 (4.07)
**Other**	
No	579 (71.39)
Yes	232 (28.61)
**Pregnancy complication**	
No	657 (81.01)
Yes	154 (18.99)

ART: assisted reproductive technology; AMA: advanced maternal age; IUGR: intrauterine growth retardation

The overall procedure-related complication rate was 3.83% (31/811). By univariate logistic regression analysis, taking other risk factors into account, pregnant women with monochorionic twin pregnancies had a significantly higher rate of procedure-related complications compared to those with dichorionic twin pregnancies (8.68% vs. 2.03%, OR:4.59, 95% CI, 2.19– 9.63, *P* = 0.00). Pregnant women who underwent amniocentesis after 24^+ 0^ weeks gestation had significantly higher rate of procedure-related complications compared to those undergoing the procedure before 24 weeks gestation (7.74% vs. 2.80%, OR:2.91, 95% CI, 1.40–6.07, *P* = 0.00). Single needle insertion amniocentesis, compared to double needle insertion, was also associated with a higher rate of procedure-related complications (10.20% vs. 2.95%, OR:3.75, 95% CI, 1.71–8.21, *P* = 0.00). Additionally, twin pregnancies with polyhydramnios had significantly higher rate of procedure-related complications (11.67% vs. 3.20%, OR:4.00, 95% CI, 1.65–9.71, *P* = 0.00), whereas other indications for amniocentesis had no significant impact. The rates of procedure-related complications according to all factors under investigation and their impact on adverse pregnancy outcomes are presented in Table 2.

**Table 2:** Risk factors associated with procedure-related complications in twin pregnancies underwent amniocentesis derived by univariate logistic regression analysis

	Procedure-relate complication rate (%)	OR (95% CI)	*P* value
**Chorionicity**			
Dichorionic	2.03	1^∆^ 4.59(2.19 ~ 9.63)	< 0.001*
Monochorionic	8.68		
**Gestational age at the procedure (weeks)**			
≤ 24^+ 0^	2.80	1^∆^ 2.91(1.40 ~ 6.07)	< 0.001*
> 24^+ 0^	7.74		
**Conceived way**			
ART	3.24	1^∆^ 1.48(0.72 ~ 3.05)	0.28
Spontaneous	4.73		
**Numbers of needle insertions**			
2	2.95	1^∆^ 3.75(1.71 ~ 8.21)	< 0.001*
1	10.20		
**Hemorrhage during this pregnancy**			
No	3.61	1^∆^ 2.43(0.70 ~ 8.39)	0.16
Yes	8.33		
**Nulliparity**			
Yes	4.23	1^∆^ 0.59(0.22 ~ 1.57)	0.29
No	2.55		
**Number of miscarriages in the 1st trimester**			
< 2	4.09	1^∆^ 0.69(0.26 ~ 1.81)	0.45
≥ 2	2.84		
**History of miscarriage in the 2nd trimester**			
No	3.71	1^∆^ 1.85(0.42 ~ 8.15)	0.41
Yes	6.67		
**Stained amniotic fluid**			
No	3.64	1^∆^ 1.35(0.54 ~ 3.35)	0.52
Yes	4.84		
**Indication**			
**Age**			
Non-AMA	4.70	1^∆^ 0.40(0.15 ~ 1.06)	0.06
AMA	1.94		
**Structural Abnormality**			
No	3.74	1^∆^ 1.08(0.5 ~ 2.324)	0.85
Yes	4.02		
**Polyhydramnios**			
No	3.20	1^∆^ 4.00(1.65 ~ 9.71)	< 0.001*
Yes	11.67		
**IUGR**			
No	3.60	1^∆^ 2.68(0.77 ~ 9.31)	0.12
Yes	9.09		
**Other**			
No	4.32	1^∆^ 0.59(0.24 ~ 1.45)	0.25
Yes	2.59		
**Pregnancy complication**			
No	4.11	1^∆^ 0.62(0.21 ~ 1.81)	0.38
Yes	2.60		

^∆^: Indicates reference category

*: *P* < 0.05

OR, odds ratio; CI, confidence interval; ART: assisted reproductive technology; AMA: advanced maternal age; IUGR: intrauterine growth retardation

To adjust for potentially confounding effects, a multivariate stepwise logistic regression analysis was conducted with procedure-related complications as dependent variables. Monochorionic pregnancy (aOR 4.06, 95% CI, 1.92–8.59), gestation age at procedure > 24^+ 0^ weeks (aOR 2.76, 95% CI, 1.30–5.87), and single-needle insertion (aOR 3.26, 95% CI, 1.45–7.31) were still statistically significant, whereas polyhydramnios had no significant impact (Table 3). 

**Table 3:** Significant risk factors associated with procedure-related complications in twin pregnancies underwent amniocentesis derived by multivariate logistic regression analysis

	aOR (95% CI)	*P* value
**Chorionicity**		
Dichorionic	1^∆^ 4.06(1.92 ~ 8.59)	< 0.001*
Monochorionic		
**Gestational age at the procedure (weeks)**		
≤ 24^+ 0^	1^∆^ 2.76(1.30 ~ 5.87)	0.008*
> 24^+ 0^		
**Numbers of needle insertions**		
2	1^∆^ 3.26(1.45 ~ 7.31)	0.004*
1		

^∆^: Indicates reference category

*: *P* < 0.05

aOR, adjusted odds ratio; CI, confidence interval

Further, the procedure-related complications after amniocentesis in dichorionic and monochorionic twins were analyzed separately. In dichorionic twin pregnancy, pregnant women who underwent amniocentesis after 24^+ 0^ gestation weeks had significantly higher rate of procedure-related complications compared to those undergoing the procedure before 24 weeks gestation (OR 4.47, 95% CI, 1.14–14.14, *P* = 0.01). In monochorionic twin pregnancy, numbers of needle insertions (aOR 3.15, 95% CI, 1.05–9.45, *P* = 0.04), hemorrhage during this pregnancy (aOR 12.01, 95% CI, 2.41–59.85, *P* = 0.00) and polyhydramnios (aOR 5.03, 95% CI, 1.50–16.89, *P* = 0.01) may affect the rate of procedure-related complication. (Supplementary Tables 1 and 2)

Besides, we subdivided the group into procedure before 24 weeks gestation and after 24 weeks. In the group before 24 weeks, the chorionicity (aOR 5.14, 95% CI, 1.85–14.24, *P* = 0.00) and numbers of needle insertions (aOR 3.76, 95% CI, 1.36–10.37, *P* = 0.01) were risk factors of procedure-related complications. In the group after 24 weeks, there were no definite risk factor (*P>0.05*). (Supplementary Tables 3 and 4)

## Discussion

Procedure-related complications after invasive tests are a major concern for pregnant women. Complications of amniocentesis in twin pregnancies may be higher than those recently reported for singleton pregnancies [17, 18]. Published reports have shown a wide range of fetal loss rates due to different definitions of fetal loss. These studies also varied in the procedures performed at different gestations. Millaire et al. reported that compared with women unexposed to the procedure, amniocentesis increased the risk of fetal losses prior to 20 to 24 weeks’ gestation (OR 2.42, 95% CI 1.24–4.74) [19]. Similarly, Cahill et al. reported a 3.2% pregnancy loss rate before 24 weeks among 311 twin pregnancies undergoing amniocentesis [20]. After adjustment for advanced maternal age, chronicity, sonographic findings, alcohol exposure and race, amniocentesis was still a significant risk factor for pregnancy loss with adjusted OR 2.9 (95% CI 1.2–6.9) [20]. To date, there have been few systematic reviews of amniocentesis in twin pregnancies [15, 16, 21]. Agarwal et al. reviewed 16 published reports and found that the overall pregnancy-loss rate was 3.07% (95% CI, 1.83–4.61), while the rate of pregnancy loss before 24 weeks was 2.54% (95% CI, 1.43–3.96) [16]. Mascio et al. reviewed 16 studies and showed that the fetal loss rate before 24 weeks was 2.4% (95% CI, 1.4–3.6) [21]. Vink et al. identified 17 publications and found that the procedure-related loss rate at < 24 weeks was 3.5% (95% CI, 2.6–4.7) [15]. In the present study, a slight increase in pregnancy complication (3.83%, 31/811) was observed. The results should be interpreted cautiously. Firstly, twin pregnancies without amniocentesis were not included. Secondly, with advanced gestational age, the pregnancy complication rate naturally increased, and our study included cases with amniocentesis after 24 weeks. In our study, the complication rate of amniocentesis performed after 24 weeks was 7.74%, which is significantly higher than that performed before 24 weeks (2.80%).

However, the actual risk of procedure-related complications remains unclear. Our study determined that fetal risk is largely dependent on chorionicity. We found that monochorionic twin pregnancies were associated with a higher risk of procedure-related complications than dichorionic twin pregnancies which is in accordance with a previous study [22]. It has been widely confirmed that chorionicity and not zygosity are important determinants of perinatal mortality in twins [23]. Compared to dichorionic twins, monochorionic twins have a higher frequency of fetal and neonatal mortality and morbidities, such as fetal and congenital anomalies, prematurity, and fetal growth restriction [23]. Thus, the increased risk of procedure-related complications in monochorionic twin pregnancies could be partly due to the background risk.

Monozygotic fetuses share the same genetic material, and it is reasonable to draw amniotic fluid from one sac to represent both fetuses. However, because sonographic diagnosis of chronicity is not always perfect, and post-zygotic variations can occur, separately collecting amniotic fluid from each sac is much more reliable [24]. A recent report found that 3.8% (10/262) of monochorionic twins had discordant karyotype results [25]. Furthermore, a number of discordant aneuploidies have been reported, most of which are associated with sex chromosomes [26, 27]. In our study, amniocentesis using the double-puncture technique for each sac was recommended for both monochorionic and dichorionic twins. In practice, some individuals undergo single-needle insertion because of personal preference or the fetal position. In our univariate logistic regression analysis, a single puncture had a higher risk of procedure-related complications than double punctures, indicating that double punctures could exert a “protective” role. In fact, in our study, women with monochorionic twin pregnancies who underwent a single puncture had much higher procedure-related complication rates than those with monochorionic twin pregnancies who had double punctures. This means that the simple assumption that amniocentesis-related risks in twin pregnancies are doubled because double punctures are needed is untruthful, and double punctures are recommended even in monochorionic twin pregnancies.

Although we excluded pregnancies diagnosed with TTTs, patients with polyhydramnios, which is considered a potential risk, were not excluded. As is well known, TTTs, for which polyhydramnios is an important manifestation, is one of most serious complications with adverse perinatal outcome for monochorionic twin pregnancies. In our study, polyhydramios was a risk factor of procedure-related complications after amniocentesis in the monochorionic twin pregnancies. It means monochorionic twin pregnancy with polyhydramnios should be treated with caution. Notably, some studies have demonstrated that the degree of polyhydramnios is associated with an increased likelihood of preterm delivery, especially at < 34 weeks. However, the underlying mechanism is unclear [28, 29].

Several risk factors for adverse outcomes have been associated with amniocentesis. A large cohort study focused on the fetal loss rate up to the 24th gestational week after amniocentesis in singleton pregnancy and determined that the risk factors were maternal age, vaginal bleeding during the current pregnancy, history of second-trimester termination of pregnancy, history of first-trimester abortions, and stained amniotic fluid [12]. Compare to study in singleton pregnancy, only the hemorrhage during this pregnancy was the risk factor in the monochorionic twin group in our study. The reason for the discrepancy remains unclear. Interestingly, it is widely believed that the outcomes of ART pregnancies are less favorable than those of spontaneous pregnancies [30–32]. However, a report has shown that the conception method did not influence the pregnancy loss rate after amniocentesis in twins, and our results were consistent with this finding [33].

No study has reported the outcomes after amniocentesis of twin pregnancy in women with an advanced gestational age. Data on complication rates for amniocentesis performed after 24 weeks of gestation are sparse, even in singleton pregnancies [4, 34]. Our study comprehensively reported procedure-related complications in twin pregnancies after 24 weeks, showing increased procedure-related complication rates with increased gestational age, which is consistent with studies of singleton pregnancies [4, 34]. In the subgroup analysis, the chorionicity as well as numbers of needle insertions were risk factors of procedure-related complications when procedure before 24 gestation weeks. Whereas, there were no definite risk factor when procedure after 24 gestation weeks.

Our study has some limitations. First, without a comparison group that did not undergo amniocentesis, we could not take into consideration the background loss rate, which is associated with gestational age. Second, although most potential risk factors were considered in the analyses, a few potentially confounding factors, such as body mass index, were not considered. Third, the rate of amniocentesis exposure combined with the relatively rare outcomes of procedure-related complications limited the precision of the risk estimates. Besides, as the study adopts a retrospective and real-world design, the determination of sample size was not applicable and the limited number of positive outcomes placed our models at the risk of overfitting.

## Conclusion

To our knowledge, this is the first study to comprehensively evaluate the impact of different risk factors on procedure-related complications after amniocentesis in twin pregnancies. We demonstrated that chorionicity is the driving mechanism of complications associated with amniocentesis procedures. Comprehensive data on the risk rates and factors associated with adverse outcomes are important as genetic counselors strive to provide precise information and assist in the decision-making process of patients.

### Electronic supplementary material

Below is the link to the electronic supplementary material.

Supplementary Material 1

## Data Availability

Data and material are available on request from the corresponding author.
