# Supplementary material for: The risk factors of procedure-related complications after amniocentesis in twin pregnancies: a retrospective analysis
Source: BMC Pregnancy Childbirth. 2023 Aug 15;23:587. doi: 10.1186/s12884-023-05884-z (PMC10428564; doi:10.1186/s12884-023-05884-z)
Supplement: Supplementary file 1 — Supplementary Material 1 [file 12884_2023_5884_MOESM1_ESM.docx]

**Supplementary Table 1 Risk factors associated with** **procedure-related complications in dichorionic twin and monochorionic twin pregnancies underwent amniocentesis derived by** **univariate logistic regression analysis.**

|  | **Dichorionic** | | | **Monochorionic** | | |
| --- | --- | --- | --- | --- | --- | --- |
|  | **Procedure-relate complication rate (%)** | **OR (95% CI)** | ***P* value** | **Procedure-relate complication rate (%)** | **OR (95% CI)** | ***P* value** |
| **Gestational age at the procedure (weeks)** |  |  |  |  |  |  |
| ≤ 24^+0^ | 1.25% (6/480) | 1^∆^ |  | 5.48% (12/163) | 1^∆^ |  |
| > 24^+0^ | 5.36% (6/112) | 4.47(1.14~14.14) | 0.01* | 12.5% (7/56) | 1.80 (0.67~4.82) | 0.24 |
| **Conceived way** |  |  |  |  |  |  |
| ART | 2.3% (11/461) | 1^∆^ |  | 15.2% (5/33) | 1^∆^ |  |
| Spontaneous | 0.8% (1/131) | 0.32(0.04~2.4) | 0.27 | 7.5% (14/186) | 0.46 (0.15~1.36) | 0.16 |
| **Numbers of needle insertions** |  |  |  |  |  |  |
| 2 | 1.9% (10/533) | 1^∆^ |  | 6.1% (11/180) | 1^∆^ |  |
| 1 | 3.4% (2/59) | 1.84（0.39~8.58） | 0.44 | 20.5% (8/39) | 0.25 (0.09~0.68) | 0.01* |
| **Hemorrhage during this pregnancy** |  |  |  |  |  |  |
| No | 2.1% (12/564) | 1^∆^ |  | 7.6% (16/211) | 1^∆^ |  |
| Yes | 0% (0/28) | 0 | 0.10 | 37.5% (3/8) | 7.31 (1.6~33.41) | 0.01* |
| **Nulliparity** |  |  |  |  |  |  |
| Yes | 2.5% (12/464) | 1^∆^ |  | 9.2% (14/151) | 1^∆^ |  |
| No | 0% (0/128) | 0 | 0.10 | 7.3% (5/68) | 0.78 (0.27~2.25) | 0.64 |
| **Number of miscarriages in the 1^st^ trimester** |  |  |  |  |  |  |
| < 2 | 2.1% (10/467) | 1^∆^ |  | 9.5% (16/168) | 1^∆^ |  |
| ≥ 2 | 1.6% (2/125) | 0.74 (0.16~3.44) | 0.70 | 5.8% (3/51) | 0.59 (0.17~2.13) | 0.42 |
| **History of miscarriage in the 2^nd^ trimester** |  |  |  |  |  |  |
| No | 1.8% (11/444) | 1^∆^ |  | 8.4% (18/213) | 1^∆^ |  |
| Yes | 4.1% (1/24) | 2.20 (0.27~17.79) | 0.46 | 16.6% (1/6) | 2.17 (0.24~19.57) | 0.49 |
| **Stained amniotic fluid** |  |  |  |  |  |  |
| No | 1.9% (10/504) | 1^∆^ |  | 8.1% (15/183) | 1^∆^ |  |
| Yes | 2.2% (2/88) | 1.15 (0.25~5.33) | 0.86 | 11.1% (4/36) | 1.4 (0.44~4.49) | 0.57 |
| **Indication** |  |  |  |  |  |  |
| **Age** |  |  |  |  |  |  |
| Non-AMA | 2.5% (10/396) | 1^∆^ |  | 10.1% (16/157) | 1^∆^ |  |
| AMA | 1% (2/196) | 0.40(0.09~1.83) | 0.23 | 4.8% (3/62) | 0.45 (0.13~1.60) | 0.22 |
| **Structural Abnormality** |  |  |  |  |  |  |
| No | 2.1% (9/422) | 1^∆^ |  | 8.50% (12/140) | 1^∆^ |  |
| Yes | 1.7% (3/170) | 0.82(0.22~3.08) | 0.77 | 8.80% (7/79) | 1.04 (0.39~2.75) | 0.94 |
| **Polyhydramnios** |  |  |  |  |  |  |
| No | 1.9% (11/553) | 1^∆^ |  | 6.50% (13/198) | 1^∆^ |  |
| Yes | 2.5% (1/39) | 1.30(0.16~10.31) | 0.81 | 28.5% (6/21) | 5.70 (1.89~17.12) | 0.00* |
| **IUGR** |  |  |  |  |  |  |
| No | 1.9% (11/573) | 1^∆^ |  | 8.2 % (17/205) | 1^∆^ |  |
| Yes | 5.2% (1/19) | 2.84(0.35~23.18) | 0.33 | 14.2% (2/14) | 1.84 (0.38~8.92) | 0.45 |
| **Other** |  |  |  |  |  |  |
| No | 1.9% (8/416) | 1^∆^ |  | 10.4% (17/163) | 1^∆^ |  |
| Yes | 2.2% (4/176) | 1.19(0.35~3.99) | 0.78 | 3.50% (2/56) | 0.32 (0.07~1.42) | 0.13 |
| **Pregnancy complication** |  |  |  |  |  |  |
| No | 2.1% (10/469) | 1^∆^ |  | 9.0% (17/188) | 1^∆^ |  |
| Yes | 1.6% (2/123) | 0.76 (0.16~3.51) | 0.72 | 6.4% (2/31) | 0.69 (0.15~3.16) | 0.64 |

*: *P* < 0.05

OR, odds ratio; CI, confidence interval; ART: assisted reproductive technology; AMA: advanced maternal age; IUGR: intrauterine growth retardation

**Supplementary Table 2 Risk factors associated with procedure-related complications in monochorionic twin pregnancies underwent amniocentesis derived by multivariate logistic regression analysis.**

|  | **aOR (95% CI)** | ***P* value** |
| --- | --- | --- |
| **Hemorrhage during this pregnancy** |  |  |
| No | 1^∆^  12.01 (2.41~59.85) | 0.00* |
| Yes |  |  |
| **Polyhydramnios** |  |  |
| No | 1^∆^  5.03 (1.50~16.89) | 0.01* |
| Yes |  |  |
| **Numbers of needle insertions** |  |  |
| 2 | 1^∆^  3.15 (1.05~9.45) | 0.04* |
| 1 |  |  |

^∆^: Indicates reference category

*: *P* < 0.05

aOR, adjusted odds ratio; CI, confidence interval

**Supplementary Table 3 Risk factors associated with procedure-related complications in twin pregnancies underwent amniocentesis before or after 24 weeks derived by univariate logistic regression analysis.**

|  | **≤ 24 weeks** | | | **＞ 24 weeks** | | |
| --- | --- | --- | --- | --- | --- | --- |
|  | **Procedure-relate complication rate (%)** | **OR (95% CI)** | ***P* value** | **Procedure-relate complication rate (%)** | **OR (95% CI)** | ***P* value** |
| **Chorionicity** |  |  |  |  |  |  |
| Dichorionic | 1.3% (6/480) | 1^∆^ |  | 5.4% (6/112) | 1^∆^ |  |
| Monochorionic | 7.4% (12/163) | 6.28 (2.32~17.01) | 0.00* | 12.5% (7/56) | 2.52 (0.81~7.91) | 0.11 |
| **Conceived way** |  |  |  |  |  |  |
| ART | 2% (8/398) | 1^∆^ |  | 8.3% (8/96) | 1^∆^ |  |
| Spontaneous | 4.1% (10/245) | 2.07 (0.81~5.33) | 0.13 | 6.9% (5/72) | 0.82 (0.26~2.62) | 0.74 |
| **Numbers of needle insertions** |  |  |  |  |  |  |
| 2 | 1.9% (11/569) | 1^∆^ |  | 6.9% (10/144) | 1^∆^ |  |
| 1 | 9.5% (7/74) | 5.30(1.99~14.13) | 0.00* | 12.5% (3/24) | 1.91 (0.49~7.53) | 0.35 |
| **Hemorrhage during this pregnancy** |  |  |  |  |  |  |
| No | 2.6% (16/614) | 1^∆^ |  | 7.5% (12/161) | 1^∆^ |  |
| Yes | 6.9% (2/29) | 2.77 (0.61~12.65) | 0.19 | 14.3% (1/7) | 2.07 (0.23~18.62) | 0.52 |
| **Nulliparity** |  |  |  |  |  |  |
| Yes | 3.3% (16/478) | 1^∆^ |  | 7.3% (10/137) | 1^∆^ |  |
| No | 1.2% (2/165) | 0.35 (0.08~1.56) | 0.17 | 9.7% (3/31) | 1.36 (0.35~5.27) | 0.66 |
| **Number of miscarriages in the 1^st^ trimester** |  |  |  |  |  |  |
| < 2 | 3% (15/498) | 1^∆^ |  | 8% (11/137) | 1^∆^ |  |
| ≥ 2 | 2.1% (3/145) | 0.68 (0.19~2.38) | 0.55 | 6.5% (2/31) | 0.79 (0.17~3.76) | 0.77 |
| **History of miscarriage in the 2^nd^ trimester** |  |  |  |  |  |  |
| No | 2.8% (17/618) | 1^∆^ |  | 7.4% (12/163) | 1^∆^ |  |
| Yes | 4% (1/25) | 1.47 (0.19~11.53) | 0.71 | 20% (1/5) | 3.15 (0.33~30.41) | 0.32 |
| **Stained amniotic fluid** |  |  |  |  |  |  |
| No | 2.6% (14/537) | 1^∆^ |  | 7.3% (11/150) | 1^∆^ |  |
| Yes | 3.8% (4/106) | 1.46 (0.47~4.54) | 0.51 | 11.1% (2/18) | 1.58 (0.32~7.77) | 0.57 |
| **Indication** |  |  |  |  |  |  |
| **Age** |  |  |  |  |  |  |
| Non-AMA | 3.5% (14/405) | 1^∆^ |  | 8.1% (12/148) | 1^∆^ |  |
| AMA | 1.7% (4/238) | 1.49 (0.55~4.04) | 0.43 | 5% (1/20) | 1.32 (0.9~1.93) | 0.15 |
| **Structural Abnormality** |  |  |  |  |  |  |
| No | 2.5% (12/480) | 1^∆^ |  | 11% (9/82) | 1^∆^ |  |
| Yes | 3.7% (6/163) | 3.62 (0.44~29.88) | 0.23 | 4.7% (4/86) | 1.23 (0.25~5.95) | 0.80 |
| **Polyhydramnios** |  |  |  |  |  |  |
| No | 2.4% (15/620) | 1^∆^ |  | 6.9% (9/131) | 1^∆^ |  |
| Yes | 13% (3/23) | 6.05 (1.62~22.58) | 0.01* | 10.8% (4/37) | 1.64 (0.48~5.67) | 0.43 |
| **IUGR** |  |  |  |  |  |  |
| No | 2.7% (17/632) | 1^∆^ |  | 7.5% (11/146) | 1^∆^ |  |
| Yes | 9.1% (1/11) | 0.5 (0.16~1.53) | 0.22 | 9.1% (2/22) | 3.15 (0.33~30.41) | 0.32 |
| **Other** |  |  |  |  |  |  |
| No | 3.3% (14/429) | 1^∆^ |  | 7.3% (11/150) | 1^∆^ |  |
| Yes | 1.9% (4/214) | 0.56 (0.18~1.74) | 0.57 | 11.1% (2/18) | 1.58 (0.32~7.77) | 0.58 |
| **Pregnancy complication** |  |  |  |  |  |  |
| No | 3.3% (17/520) | 1^∆^ |  | 7.3% (10/137) | 1^∆^ |  |
| Yes | 0.8% (1/123) | 0.24 (0.03~1.84) | 0.24 | 9.7% (3/31) | 1.36 (0.35~5.27) | 0.66 |

^∆^: Indicates reference category

*: *P* < 0.05

OR, odds ratio; CI, confidence interval; ART: assisted reproductive technology; AMA: advanced maternal age; IUGR: intrauterine growth retardation

**Supplementary Table 4 Risk factors associated with procedure-related complications in twin pregnancies underwent amniocentesis before 24 weeks derived by** **multivariate logistic regression analysis.**

|  | **aOR (95% CI)** | ***P* value** |
| --- | --- | --- |
| **Chorionicity** |  |  |
| Dichorionic | 1^∆^  5.14(1.85~14.24) | 0.00* |
| Monochorionic |  |  |
| **Numbers of needle insertions** |  |  |
| 2 | 1^∆^  3.76(1.36~10.37) | 0.01* |
| 1 |  |  |

^∆^: Indicates reference category

*: *P* < 0.05

aOR, adjusted odds ratio; CI, confidence interval
